# Developmental Easing of Short-Term Depression in “Winner” Climbing Fibers

**DOI:** 10.3389/fncel.2019.00183

**Published:** 2019-05-01

**Authors:** Christina Pätz, Simone Brachtendorf, Jens Eilers

**Affiliations:** Carl-Ludwig-Institute for Physiology, University of Leipzig, Leipzig, Germany

**Keywords:** cerebellum, climbing fibers, Purkinje neuron, short-term plasticity, paired-pulse depression

## Abstract

The postnatal development of cerebellar climbing fiber (CF) to Purkinje neuron (PN) synapses is characterized by a substantial pruning during the first 3 weeks after birth, switching from multiple- to single-CF innervation. Previous studies suggested that CF maturation is governed by bidirectional changes of synaptic plasticity. The strengthening of surviving “winner” CFs, which translocate from the PN soma to the dendrite, is thought to be guided by long-term potentiation (LTP), while weakening of to-be-eliminated “loser” CFs, which remain on the soma, was proposed to be due to long-term depression (LTD). However, there are conflicting results from previous studies, whether or not strengthening of winner and weakening of loser CFs during postnatal development is accompanied by changes in short-term plasticity and, thus, whether pre- or postsynaptic forms of LTD and LTP are operational. We, therefore, analyzed the developmental profile of paired-pulse depression (PPD) in “weak” and “strong” CFs in 3–21-day old *Igsf9*-eGFP mice, which allow visual identification of GFP-labeled CFs. We found that in 3–8-day old mice strong CFs are marked by a stronger PPD compared to weak CFs. Surprisingly, PPD of strong CFs eases during maturation, while PPD in weak CFs remains unchanged. This easing of PPD is neither due to changes in presynaptic influx-release coupling nor to an increased saturation of postsynaptic receptors. Thus, our results imply that synaptic contacts of CFs show distinct features of PPD depending on their affiliation to winner or loser CFs and depending on their somatic or dendritic location.

## Introduction

During postnatal maturation of the central nervous system, synaptic contacts face diametrically opposed fates: if they are part of a successfully formed neuronal circuit, they will mature into well-established, strengthened contacts that may persist for the rest of the individual’s life; synaptic contacts not contributing to meaningful neuronal circuits are doomed to become disintegrated or “pruned” within a period of days to weeks. How such “winner” and “loser” synaptic contacts are initially selected and subsequently strengthened or pruned, respectively, is only partly understood (Schuldiner and Yaron, 2015; Piochon et al., 2016).

The cerebellar climbing fiber (CF) to Purkinje neuron (PN) synapse is a well suited model system for studying winner and loser synapses in rodents (Hashimoto and Kano, 2003; Kano et al., 2018). At birth, each PN is contacted by several CFs that form preliminary synaptic contacts on the soma and perisomatic processes of the immature PN (Altman, 1972). In parallel to the maturation of the PN, during which the perisomatic processes are retracted, a single winner CF forms synaptic contacts on the growing main apical dendrite, while all other CFs stay confined to the PN soma and are ultimately eliminated (Kano et al., 2018), leading to a CF monoinnervation in at least 98% of the PNs (Eccles et al., 1966). Both processes, the strengthening of winner CFs as well as the elimination of loser CFs, have been linked to long-term plasticity. Long-term potentiation (LTP) has been shown to occur at winner synapses only (Bosman et al., 2008; Ohtsuki and Hirano, 2008), either as presynaptic LTP associated with an increase in paired-pulse depression (PPD; Ohtsuki and Hirano, 2008), or, as postsynaptic LTP not associated with alterations in PPD (Bosman et al., 2008). Long-term depression (LTD), on the other hand, has been reported to occur at loser synapses only, expressed presynaptically with a decrease in PPD (Ohtsuki and Hirano, 2008). Based on these findings, it could be expected that winner and loser CFs are characterized by distinct developmental profiles, in which loser CFs show a PPD that decreases with age (Ohtsuki and Hirano, 2008) while winner CFs show either an increasing (Ohtsuki and Hirano, 2008) or stable (Bosman et al., 2008) PPD. The previous finding that loser CFs show stronger PPD compared to winner CFs (Hashimoto and Kano, 2003) is seemingly in conflict to this hypothesis, though, this study did not discriminate for postnatal age.

Here, we analyzed the developmental profile of PPD in weak and strong CFs during the first 3 weeks after birth. We found that in the first postnatal week strong CFs showed a stronger PPD compared to weak CFs and that during the following two postnatal weeks PPD in weak CFs remained unchanged, while strong CFs, unexpectedly, showed a developmental easing of PPD. We further show that the changes in PPD of strong CFs are not due to alterations in the influx-release coupling and cannot be attributed to an increase in receptor saturation. Thus, winner CFs belong to the group of synaptic connections that undergo a developmental reduction in PPD (reviewed in Feldmeyer and Radnikow, 2009), contrasting the developmental increase seen, for example, in parallel fiber to PN synapses (Baur et al., 2015). Further on, our data are in line with the hypothesis that somatic CF contacts undergo presynaptic LTP (Ohtsuki and Hirano, 2008), while CFs that translocated to the PN dendrite may undergo postsynaptic forms of plasticity (Hansel and Linden, 2000; Bosman et al., 2008).

## Materials and Methods

### Ethics Statement

Animal experiments were performed in accordance with the EU Directive 2010/63/EU and were approved by the State directorate of Saxony, Germany.

### Animals and Slice Preparation

Experiments were performed in cerebellar slices from transgenic *Igsf9*-eGFP mice (Gensat, Rockefeller University, New York, NY; RRID:MMRRC_030804-UCD) from postnatal day (P) 3–21, without considering gender. Breeding and genotyping was done as described previously (Pätz et al., 2018).

Mice were decapitated under deep isoflurane (Baxter GmbH, Unterschleißheim, Germany) anesthesia, the brain was rapidly excised and placed in ice-cold artificial cerebrospinal fluid (ACSF) consisting of (in mM): 20 Glucose, 125 NaCl, 2.5 KCl, 1.25 NaH_2_PO_4_, 26 NaHCO_3_, 2 CaCl_2_ and 1 MgCl_2_, saturated with carbogen (95% O_2_, 5% CO_2_), pH 7.4. Sagittal slices (200 μm) were cut with a microtome (HM 650 V, Microm, Walldorf, Germany), kept for 45 min at 35°C, and subsequently stored at room temperature (19–20°C) in ACSF. Recordings were performed at room temperature. Unless specified otherwise, all chemicals were obtained from Sigma-Aldrich, Seelze, Germany.

### Electrophysiology

Recordings of extracellular postsynaptic currents (EPSCs) from whole-cell patch-clamped PNs, evoked by targeted stimulation of visually identified GFP-labeled CFs, were performed as in Pätz et al. (2018) from PNs independent on their location in a specific lobule. Patch pipettes were prepared from borosilicate glass (Hilgenberg, Malsfeld, Germany) with a PC-10 puller (Narishige, Tokyo, Japan), having resistances of 5–6 MΩ when filled with pipette solution, which contained (in mM): 147 CsMeSO_3_, 4 NaCl, 4 Mg-ATP, 0.4 GTP, 10 HEPES, 1.6 MgCl_2_, 0.5 EGTA and 50 μM Atto 594 (Atto-Tec, Siegen, Germany) dissolved in purified water with the pH titrated to 7.3 using CsOH. Slices were transferred to the recording chamber and continuously perfused at 3 ml/min with ACSF supplemented with 10 μM gabazine to block spontaneous GABAergic currents and, unless otherwise denoted (data shown in Figure 4), with a submaximal concentration (1 mM) of the rapid glutamate receptor antagonist kynurenic acid (KYN) to allow proper voltage clamping of CF responses and to minimize effects of receptor saturation (Wadiche and Jahr, 2001; Foster and Regehr, 2004). Before each recording, the series resistance was automatically compensated to reach a remaining, uncompensated series resistance of 10 MΩ. The offset potential was corrected for the liquid junction potential (14 mV). Data acquisition and analysis was done using Patchmaster software (version 2x90.1, HEKA Electronik, Lambrecht, Germany; RRID:SCR_000034).

Whole-cell patch-clamp recordings from PNs and targeting of GFP-positive (GFP^+^) CFs was performed under visual control using an Olympus FV1000 two-photon laser-scanning microscope (Olympus, Tokyo, Japan) equipped with a Mai Tai DeepSee laser (Spectra-Physics, Darmstadt, Germany) set to a center wavelength of 915 nm. Epifluorescence signals were acquired using an 40×/0.8 NA water-immersion objective, a 570 nm dichroic mirror, and 495–540 nm and 575–630 nm emission filters (for the GFP and Atto 594 signals, respectively); transmitted light was detected with a PMT-based detector (Olympus). Targeted stimulation of GFP-labeled CFs was done as follows: (1) scanning for regions with clearly visible individual GFP^+^ CFs, (2) establishing a somatic whole-cell patch with a nearby PN, which is thereby dialyzed with the red fluorescent dye Atto 594, and (3) using transmitted light imaging together with electronically overlaid red and green fluorescence to place a glass pipette (10 MΩ, filled with ACSF) in close proximity to a GFP^+^ CF projecting to the patched PN (Pätz et al., 2018).

Paired stimuli were applied at 100 ms inter-stimulus interval (ISI) and CFs were identified by PPD and all-or-none responses of the first response, yielding a step-wise stimulus-response curve (SRC) at increasing stimulus strength (Eccles et al., 1966; Konnerth et al., 1990). When using multiple electrodes for stimulating several CFs, the selective activation of individual CFs was assured as described in Bosman et al. (2008) and Pätz et al. (2018). CF inputs were categorized as “weak” when their EPSC peak amplitudes (at a holding potential of –75 mV and in 1 mM KYN) remained below a threshold of 360 pA and “strong” otherwise.

The coupling between Ca^2+^ influx and release was characterized by application of exogenous chelators (Adler et al., 1991) as their membrane-permeant acetoxymethyl-ester (AM) variants. Following a control period of 10 min, either 10 μM of BAPTA- or EGTA-AM (both from Invitrogen, Eugene, OR), dissolved in 0.1% DMSO and 0.01% pluronic, were applied for 15 min, followed by a 10 min wash-out phase. Control recordings were performed with application of just DMSO and pluronic.

To analyze the dose-dependent effect of KYN on CF-EPSC amplitudes and their paired-pulse ratios (PPRs, Figure 4), first and second CF responses to paired stimuli (100 ms ISI) were recorded in slices perfused with normal ASCF followed by application of increasing KYN concentrations (0.05, 0.2, 1 and 2 mM) for 10 min each. SRCs were acquired prior application of KYN and after each incubation interval in order to ascertain stimulation of a single CF.

### Statistical Analyses

**FIGURE 1:**
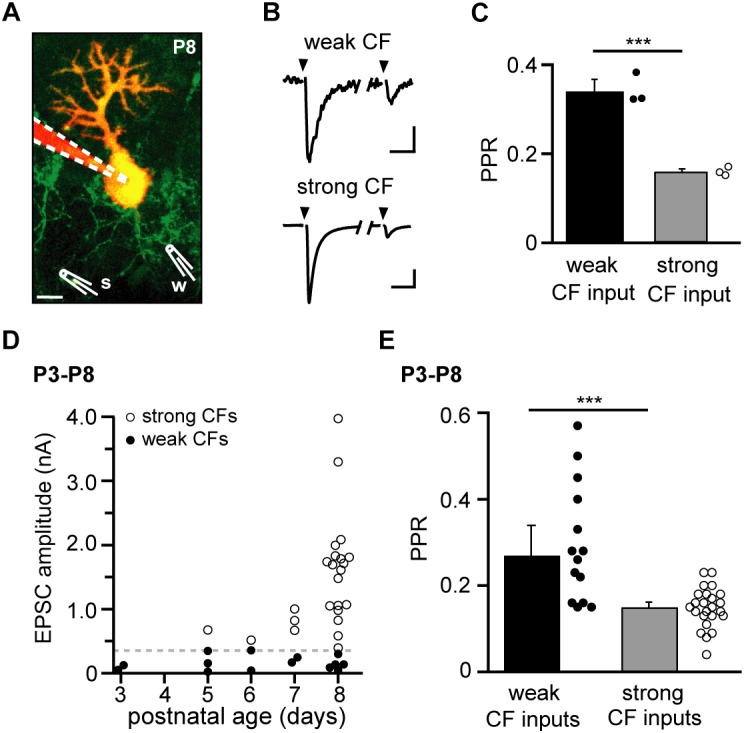
Deepened depression in “strong” compared to “weak” climbing fiber (CF) inputs in the immature cerebellum. **(A)** Two-photon image of a Purkinje neuron (PN) loaded with a red indicator dye via a somatic whole-cell patch pipette (dashed lines) in an acute cerebellar slice from an 8-day-old (P8) *Igsf9*-eGFP mouse, in which CFs express GFP (green). Two independent stimulation electrodes (schematically drawn and labeled “w” and “s” for “weak” and “strong” CF, respectively) were placed on visually identified CFs. Projection of 37 images obtained with 1 μm z-interval; scale bar 10 μm. **(B)** Paired-pulse behavior (100 ms inter-stimulus interval) of excitatory postsynaptic currents (EPSCs) of the weak and strong CF, respectively. The stimulation of individual CFs was ascertained by an all-or-none behavior in stimulus-response curves (SRCs). Traces are averages of 11 (“weak”) and 12 (“strong”) successful CF stimulations performed in 1 mM kynurenic acid. Scale bars are 10 pA and 10 ms (“weak CF”) and 0.2 nA and 20 ms (“strong CF”); same experiment as illustrated in panel **(A)**. **(C)** Paired-pulse ratios (PPRs) from three successive SRCs [from which successful responses are shown averaged in panel **(B)**]. Filled and open circles represent PPRs from single SRCs (average of 2–6 responses) in the weak and strong CF, respectively. Bar graphs represent mean + SEM. Note that the strong CF showed a significantly smaller PPR compared to the weak CF (*P* ≤ 0.001^∗∗∗^). **(D)** EPSC amplitudes of weak and strong CFs (filled and open circles, respectively) vs. age (P3–P8; defined as age group “young”). The dashed line represents the chosen threshold (360 pA) separating weak and strong CFs (*n* = 14 and 24 for weak and strong CFs, respectively). **(E)** PPRs of weak and strong CFs (filled and open circles, respectively), same data as in panel **(D)**. Bar graphs show median + SEM of median (*P* ≤ 0.001^∗∗∗^; *n* = 14 and 24 for weak and strong CFs, respectively, including *n* = 5 PNs in which a weak and a strong CF were recorded simultaneously).

**FIGURE 2:**
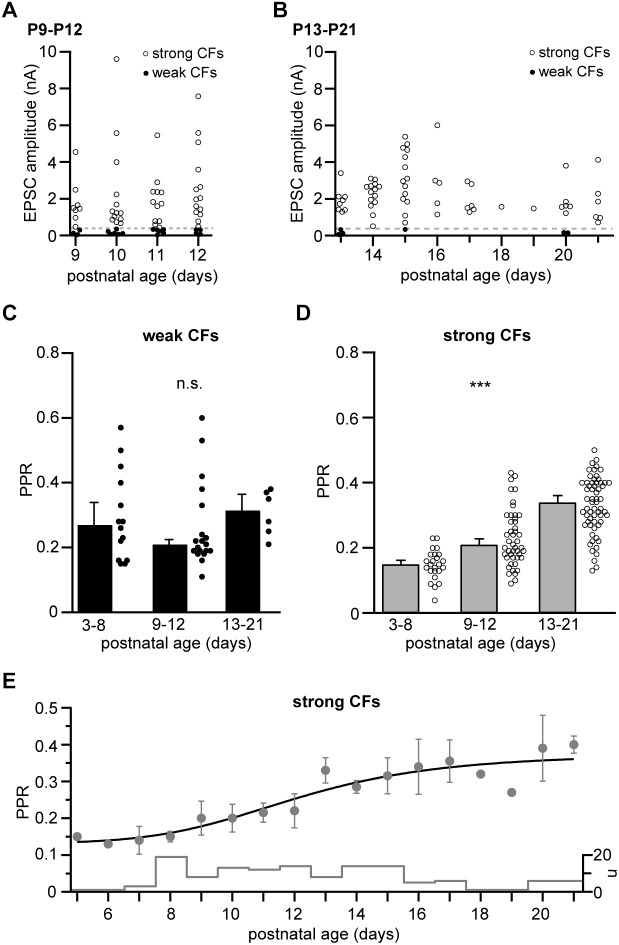
Strong, not weak CFs undergo a developmental easing of depression. **(A)** EPSC amplitudes of weak and strong CFs (filled and open circles, respectively) recorded in mice of the “transient” age group (P9–P12, *n* = 19 and 47 for weak and strong CFs, respectively, including *n* = 6 PNs in which two CFs were recorded simultaneously). The dashed line marks the threshold separating weak and strong CFs (360 pA). **(B)** Same as in panel **(A)** but showing CF-EPSC amplitudes in “young adult” mice (P13–P21, *n* = 6 and 61 for weak and strong CFs, respectively, including *n* = 1 PN in which two CFs were recorded simultaneously). Note that weak CFs were barely found in this age group. **(C,D)** Comparison of PPRs of weak **(C)** and strong **(D)** CFs pooled per age group (“young” = P3–P8, “transient” = P9–P12, and “young adult” = P13-P21). Data are shown as median + SEM of median (bars) and PPRs of individual CFs (circles). Note that PPRs of weak CFs remain unchanged (Kruskal–Wallis test, *P* = 0.319), while PPRs of strong CF inputs significantly increase during postnatal development [Kruskal–Wallis test, *P* ≤ 0.001^∗∗∗^ with *P* < 0.05 for all pairwise comparisons (Dunn’s test)]. **(E)** Median ± SEM of median values for PPRs of strong CFs vs. postnatal age. The line represents a sigmoidal fit to the data, yielding an inflection point at P12. The “cityscape” graph shows the number of CFs (*n*) included per age.

**FIGURE 3:**
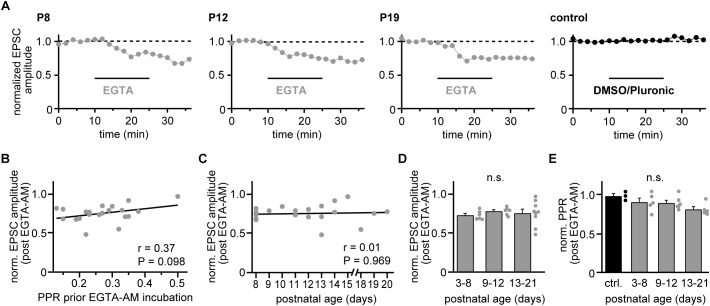
Coupling distances remain stable during development. **(A)** Examples of recordings from representative strong CF-PN connections showing EGTA-AM experiments (gray) in young, transient age, and young adult mice (P8, P12, and P19, respectively), as well as a control recording (black, P11) at 2 min binning (0.1 Hz stimulation). Experiments comprised 10 min baseline recording, 15 min incubation with 10 μM EGTA-AM or control solution (pluronic in DMSO) and 10 min washout. Data are normalized to their mean during the control period. Note the similar effectiveness of EGTA-AM at the different age stages. **(B)** EGTA-induced reduction of EPSC amplitudes (average of washout period) vs. PPR values prior EGTA-AM incubation. Each point represents data from a single cell (*n* = 21). Note that the EGTA effect shows no correlation to the initial PPR (line, *r* = 0.37, *P* = 0.098). **(C)** Reduction of CF-EPSC amplitudes following EGTA-AM incubation vs. postnatal age [same experiments as illustrated in panel **(B)**]. Note the absence of a correlation between EGTA-AM effectiveness and age (line, *r* = 0.01, *P* = 0.969). **(D)** Data shown in panel **(C)** grouped into data from young (P3–P8), transient (P9–P12) and young adult (P13–P21) animals. Bars show mean + SEM and dots represent the data from individual cells (n.s.: *P* = 0.391, Kruskal–Wallis test). **(E)** EGTA-induced changes in PPR (normalized to their values prior to EGTA application) vs. postnatal age group (gray) and control recordings (black). Dots represent data from individual cells, bars the respective mean + SEM values (n.s.: *P* = 0.103, One Way ANOVA).

**FIGURE 4:**
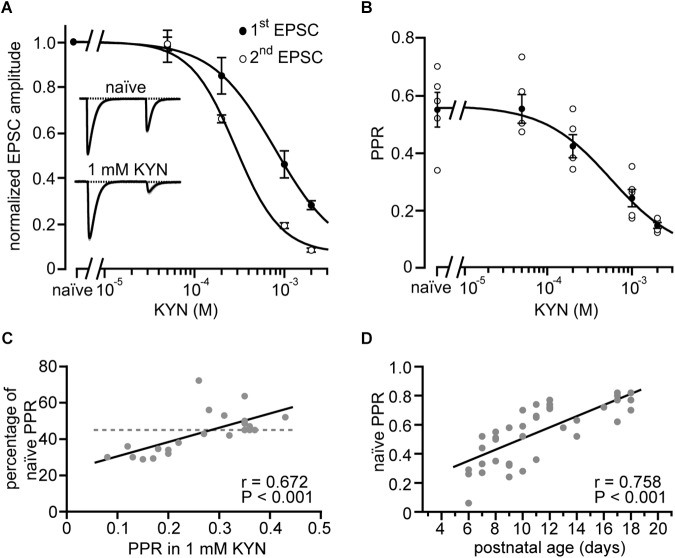
Easing of receptor saturation accompanies developmental easing of depression. **(A)** CF-EPSC amplitudes of the first and second response (closed and open circles, respectively) of paired stimulation vs. the concentration of bath-applied kynurenic acid (KYN). Data are from strong CF synapses and were normalized to their values in the absence of KYN (“naïve”) and are shown as mean ± SE (*n* = 5 recordings, P11–P16). Lines represent fits to Hill equations to the data. Note that KYN has a stronger effect on the second CF-EPSC amplitude compared to the first (cf. Wadiche and Jahr, 2001; Foster and Regehr, 2004). Insets show self-normalized example EPSCs recorded in absence (top) and presence of 1 mM KYN (bottom) from a representative CF-PN connection from a P13 mouse. **(B)** Paired-pulse ratios of CFs plotted vs. KYN concentration. Shown are the data of individual cells [open circles, same as in panel **(A)**], their mean ± SE (filled circles), and a fit to a Hill equation (line) to the mean values that yielded a half-maximal effect at 570 μM KYN. **(C)** Reduction of PPR [PPR in 1 mM KYN divided by PPR in the absence of KYN (“naïve”), expressed in percent] plotted vs. PPR in 1 mM KYN. Each point (*n* = 22) represents a single cell. The dashed line represents the average reduction of PPR by 1 mM KYN as calculated from panel **(B)**. Note that PPRs measured in KYN correlate with the effectiveness of KYN on PPR (line, *r* = 0.672, *P* < 0.001^∗∗∗^). **(D)** CF-PPRs recorded in the absence of KYN (“naïve”) show a linear correlation (*n* = 41, line, *r* = 0.758, *P* < 0.001^∗∗∗^) to postnatal age. Note that easing of depression is observed also in the absence of KYN.

Statistics were performed with Sigma Plot 11.0 (Systat Software, San Jose, CA; RRID:SCR_003210). Normal distribution of data was tested using the “Shapiro–Wilk normality test” and statistical significance was analyzed via a Rank sum test for two groups (Figure 1C,E), “Kruskal–Wallis one way analysis of variance on ranks” for three groups (Figure 2C,D, 3D) followed by the “Dunn–Holland–Wolfe Test” in case a significant difference in the median values was reported (Figure 2D) or, in case of normal distribution and equal variances, a One Way ANOVA (Figure 3E). A *P* value ≤ 0.05 was considered to be statistically significant (^∗∗∗^ denotes *P* ≤ 0.001). Data correlation was tested in Igor Pro 6.37 Software (Wavemetrics, Lake Oswego, OR; RRID:SCR_000253) via “Pearson product moment correlation” (Figure 3B,C, 4C,D); relationships between variables were considered to be statistically significant at *P* ≤ 0.05.

## Results

### Differences in Synaptic Short-Term Depression Between “Weak” and “Strong” CFs at P3–P8

We recorded CF-evoked EPSCs in whole-cell patch-clamped PNs of *Igsf9*-eGFP mice, which express GFP in CFs and, thereby, allow visual identification of individual CFs innervating a given PN (Pätz et al., 2018). After dialyzing the patched PN with a red indicator dye (Atto 594) and searching for optimal locations for stimulating individual GFP^+^ fibers using two-color two-photon microscopy, stimulation electrodes were positioned under visual control (using a transmitted light detector) directly on the selected CFs (Figure 1A). Specificity of stimulation was ascertained by testing for step-wise SRCs (not shown; Konnerth et al., 1990) and, in experiments with more than one stimulation electrode, for the absence of PPD in cross-wise stimulations (not shown; Bosman et al., 2008).

The different CFs innervating a given PN typically evoked EPSCs with varying amplitudes, allowing distinguishing “weak” and “strong” inputs in a given PN (Figure 1B, peak amplitudes of 36 and 820 pA, respectively, for the first response). Also the paired-pulse ratio (PPR, 100 ms stimulus interval) of CFs innervating a given PN differed. In the example illustrated in Figure 1A,B (8-day old mouse, P8) the weak CF showed a PPR of 0.34 ± 0.03 (Mean ± SEM, *n* = 3 repetitions) while the strong CF showed a PPR of 0.16 ± 0.01 (Figure 1C). As described previously (see, for example, Hashimoto and Kano, 2003; Bosman et al., 2008), the disparity in CF amplitudes grew with postnatal age (Figure 1D). In order to compare PPR values across different cells and ages, we grouped CF inputs according to whether their EPSCs (first response) reached a level of 360 pA or not (“strong” and “weak” inputs, respectively; dashed gray line in Figure 1D). Notably, this grouping allowed inclusion of experiments in which only a single CF could be identified per PN and experiments, in which no “strong” GFP^+^ CF could be identified. In the age group “young” (defined as P3–P8 in this study), weak and strong CFs showed a statistically significant difference in PPR. Similar to the example illustrated in Figure 1A–C, weak CFs had, on average, PPRs of 0.27 ± 0.07 (Median ± SEM median, *n* = 14 cells) while strong CFs had PPRs of 0.15 ± 0.01 (*n* = 24 cells; *P* ≤ 0.001^∗∗∗^; Figure 1E). Taking changes in PPR as a surrogate for changes in presynaptic release probability (Fioravante and Regehr, 2011), this differences would be in accordance with the idea that presynaptic forms of LTP and LTD underlie the establishment of “winner” and elimination of “loser” CF inputs, respectively (Ohtsuki and Hirano, 2008).

### Developmental Easing of Paired-Pulse Depression in Strong CF Inputs

We next analyzed CF responses in older mice, i.e., in later phases of CF elimination and maturation (Kano and Hashimoto, 2009). To this end, we defined the age groups “transient” (P9–P12) and “young adult” (P13–P21), age spans roughly corresponding to the “early” and “late” phases of CF elimination, as defined by Kano and Hashimoto (2009). As expected, the peak amplitudes of strong CFs continued to increase until the second postnatal week (Figure 2A,B; note the different y-scaling compared to Figure 1D) and weak CF inputs were rarely found in animals older than 2 weeks (Figure 2B). Interestingly, correlating (across all age groups) the magnitude of PPR with the corresponding EPSC amplitudes for weak and strong CFs (binned to 50 and 500 pA, respectively), revealed no significant relationship (*r* = –0.682 and 0.326, *P* = 0.091 and 0.277 for weak and strong CFs, respectively; data not shown). Similarly, when comparing PPRs values of weak CFs at the different postnatal stages revealed that the PPR of weak CFs remained stable in transient and young adult animals (0.21 ± 0.01, *n* = 19 cells and 0.32 ± 0.05, *n* = 6 cells, respectively; Figure 2C), not differing from the average values in young mice (0.27 ± 0.07; *P* = 0.319, Kruskal–Wallis test). More surprisingly, PPR values in strong CFs shifted toward higher values during development with PPR values of 0.15 ± 0.01 (*n* = 24 cells) in young animals, 0.21 ± 0.02 (*n* = 47 cells) in transient age animals, and 0.34 ± 0.02 (*n* = 61 cells) in young adult animals (Figure 2D). This easing of depression was statistically highly significant (*P* ≤ 0.001^∗∗∗^, Kruskal–Wallis test, with *P* < 0.05 for all pairwise comparisons, Dunn’s test). Plotting average PPR values vs. postnatal age (Figure 2E) revealed that this easing occurred approximately in the time period between P8 and P18. This temporal profile was well described by a fit to a Hill equation with start and end PPR values of 0.13 and 0.37, respectively, a Hill coefficient of 5.03 (line in Figure 2E), and an inflexion point at P12.

### Stable Coupling in Strong CFs During Development

The observed changes in PPR values of strong CFs would be in line with a developmentally regulated change in the presynaptic release probability (Baur et al., 2015), for which corresponding changes in the Ca^2+^ influx-release coupling represent a likely mechanism (ibid.). We, therefore, tested the effectiveness of exogenous Ca^2+^ buffers on reducing EPSC amplitudes (Dittman and Regehr, 1998) in the three age groups. We bath-applied the slow Ca^2+^ buffer EGTA in its membrane-permeable form (EGTA-AM, 10 μM, dissolved in 0.1% DMSO and 0.01% pluronic) for 15 min and quantified the EPSC reduction after stable responses were obtained in the wash-out period. EGTA application led to substantial reductions in CF-EPSC amplitudes in young, transient age and young adult animals (Figure 3A) that was independent on the strength of PPD prior to EGTA application (*r* = 0.37, *P* = 0.098; Figure 3B). The effectiveness of EGTA showed no correlation with postnatal age (*r* = 0.009, *P* = 0.969; Figure 3C). Similarly, no significant differences were observed when grouping experiments into the three age groups, yielding EGTA-induced reductions of 27 ± 2%; 22 ± 4% and 24 ± 15% (mean ± SEM) in young, transient age and young adult mice (*P* = 0.391; Kruskal–Wallis test; Figure 3D). The similar effectiveness of EGTA at all postnatal stages as well as on low- and high-PPR CFs suggest that easing of depression is not due to changes in the presynaptic Ca^2+^ influx-release coupling. In agreement with the idea that the coupling distance is stable during postnatal development of strong CFs, application of EGTA did not induce age-dependent differences in strong CFs, with reductions of 10 ± 5%; 11 ± 3% and 19 ± 4% (mean ± SEM) in young, transient age, and young adult mice and 2 ± 3% in control (without EGTA-AM) recordings (*P* = 0.103; One Way ANOVA; Figure 3E).

### Easing of Depression Correlates With Reduced Receptor Saturation

Glutamate release at CF-to-PN synapses is strong enough to induce postsynaptic receptor saturation, reducing EPSC peak amplitudes and, more importantly for our study, limiting the magnitude of PPD (Wadiche and Jahr, 2001; Foster and Regehr, 2004). The so far described experiments were, therefore, conducted in the presence of the rapid glutamate receptor antagonist kynurenic acid (KYN, 1 mM), which ameliorates these effects of saturation (Wadiche and Jahr, 2001). In a final set of experiments we addressed the questions whether (i) 1 mM KYN is sufficient to cancel the effects of receptor saturation on PPR, (ii) the extent of receptor saturation correlates with PPD, and if (iii) the developmental easing of depression seen in 1 mM KYN (Figure 2D,E) can also be observed under naïve conditions (i.e., in the absence of KYN).

Figure 4A shows a semi-logarithmic plot of the dose-response relationship between CF-evoked responses in PNs to paired CF stimuli (1st and 2nd EPSC) and KYN, illustrating that KYN blocks the first EPSC significantly less than the second EPSC, and does so in a dose-dependent manner. In line with previous studies (Wadiche and Jahr, 2001; Foster and Regehr, 2004), this effect indicates that more glutamate is released during the first compared to the second stimulation, leading to stronger saturation during the first stimulation under naïve conditions. Figure 4B shows the corresponding relationship between PPR and KYN. Fitting the data to a Hill equation (line in Figure 4B) indicates that even at 2 mM KYN the PPR was still affected by receptor saturation. At this KYN concentration, however, second EPSC amplitudes were reduced to about 10% of their naïve values, hindering quantification of EPSC amplitudes from weak CF inputs. We, therefore, chose 1 mM as the standard KYN concentration in our study, a concentration not completely canceling the effects of receptor saturation on PPR (reduction to 65%; Figure 4B) but sparing enough receptors to allow for quantification of weak EPSC amplitudes (Figure 4A).

The question, whether the extent of receptor saturation correlates with the depth of PPD was addressed by plotting the PPR obtained in 1 mM KYN against the strength of the KYN effect [expressed as ratio of PPD before (naïve) and after application of 1 mM KYN; Figure 4C]. The plot revealed that CF inputs with a strong PPD (PPR < 0.24) were stronger affected by KYN than inputs with low PPD. The correlation was highly significant (*r* = 0.672, *P* < 0.001^∗∗∗^), deviating from the mean effect of KYN (44%, dashed line in Figure 4C, calculated from Figure 4B). These data indicate that immature strong CF synapses, characterized by a strong depression, release more glutamate (Hashimoto and Kano, 2003). These synapses, thereby, undergo stronger postsynaptic receptor saturation and, in turn, a stronger mitigation of their PPD (Wadiche and Jahr, 2001; Foster and Regehr, 2004).

In a final step of analysis we plotted PPD values in strong CFs obtained in the absence of KYN (“naïve”) vs. postnatal age (Figure 4D). Similar to the data obtained in 1 mM KYN (Figure 2D,E), this plot revealed the easing of depression correlates strongly with postnatal age (*r* = 0.758, *P* < 0.001^∗∗∗^). Naïve weak CFs, on the other hand, showed no correlation between PPD and age (P5–P12; median per postnatal day; *r* = 0.626, *P* = 0.097, data not shown), which, similar to the results obtained in 1 mM KYN (Figure 2C), suggests that PPD in weak CFs remains unchanged during postnatal development. Thus, the developmental stability of depression in weak CFs and the developmental easing of depression in strong CFs is neither due to nor counterbalanced by relief of saturation, but can be observed under naïve conditions.

## Discussion

Our results reveal unexpected developmental differences in short-term plasticity of “weak” and “strong” CFs: (i) in the first postnatal week PPD is stronger in strong CFs compared to weak CFs, (ii) in weak CFs PPD remains stable during the second and third postnatal week, while (iii) strong CFs undergo an easing of PPD during maturation. This easing is neither due to changes in presynaptic influx-release coupling nor to an increase in saturation of postsynaptic receptors. These results significantly extend a previous study by Hashimoto and Kano (2003), reporting on a stronger PPD in weak compared to strong CFs, in which data were pooled mostly from P10 to P14. Since the data were not differentiated regarding the exact age, this pooling may have obscured the unexpected developmental profile of PPD in CFs (Figure 2E).

In this study, we used the transgenic mouse line *Igsf9*-eGFP expressing GFP in a substantial fraction of CFs. We previously showed that this line allows studying CF-specific responses by visually guided stimulation of GFP-labeled CFs (Pätz et al., 2018). Targeted stimulation simplified recordings of individual CF inputs converging on immature, multiple innervated PNs (Figure 1A). More importantly for the present study, this approach allows identification of CFs independent on whether or not they show PPD, the electrophysiological hallmark of mature CFs (Konnerth et al., 1990). It had previously been reported that weak CFs can undergo a presynaptic form of LTD and an associated easing of PPD, resulting even in a paired-pulse potentiation (Ohtsuki and Hirano, 2008), the hallmark of parallel fiber (PF) to PN synapses (Konnerth et al., 1990). Without visual identification, such “atypical” CFs could not have been included in our analysis. However, we did not observe that any CF, weak or strong, did not show depression (but see Ohtsuki and Hirano, 2008).

Previous studies showed that weak CFs can undergo LTD, associated with an easing of PPD (Ohtsuki and Hirano, 2008), while strong CFs can undergo LTP, associated (Ohtsuki and Hirano, 2008) or not associated (Bosman et al., 2008) with an enhancement of PPD. These findings suggested that elimination of surplus “loser” CFs and strengthening of remaining “winner” CFs occurs in response to LTD and LTP, respectively (Bosman et al., 2008; Ohtsuki and Hirano, 2008) and that the magnitude of PPD would be a measure of the ongoing elimination or strengthening. Our data on the developmental profile of PPD in weak and strong CFs are not directly compatible with this hypothesis, since neither weak CFs did change their PPD (Ohtsuki and Hirano, 2008) nor did strong CFs show a constant (Bosman et al., 2008) or strengthened PPD (Ohtsuki and Hirano, 2008).

For weak CFs, our data can be reconciled with the hypothesis that LTD indeed underlies their elimination (Piochon et al., 2016) by assuming that synaptic homeostasis counterbalances LTD (Weyhersmüller et al., 2011). In this scenario, weak CFs may be susceptible to LTD, which lasts at least 30 min and is of presynaptic origin (Ohtsuki and Hirano, 2008); over hours and days, however, synaptic homeostasis would revoke the depression, resulting in the stable PPD of weak CFs we observed throughout development. The final elimination of surplus CFs may then occur by an exhaustion of the homeostatic processes or, alternatively, by a developmentally regulated termination of homeostasis in CFs.

For strong CFs, besides possible molecular aspects of maturation (Kusch et al., 2018), the topology of their synapses needs to be taken into consideration (Altman, 1972; Hashimoto et al., 2009). Strong CFs, as weak ones, contact PNs initially exclusively on their somata and perisomatic processes, which are present before PNs develop their main dendritic tree (Altman, 1972). In contrast to weak CFs, strong CFs begin to form dendritic synapses as soon as PNs evolve their apical dendritic tree, leading to an almost exclusively dendritic innervation by P15 (Hashimoto et al., 2009; Carrillo et al., 2013). Thus, depending on the postnatal stage, there are two types of strong CF synapses: somatic ones (declining in number) and dendritic ones (increasing in number). The developmental easing of PPD of strong CFs (Figure 2E) would be in line with the hypothesis that somatic CF synapses, competing with neighboring weak CFs, are indeed strengthened by presynaptic LTP, undergoing a concomitant increase in PPD (Ohtsuki and Hirano, 2008). Dendritic synapses of the same fibers, not competing with neighboring weak CFs, may represent mature-type CF synapses, which continue to grow, i.e., form more synaptic contacts on the growing dendritic tree, but become susceptible to LTD (Hansel and Linden, 2000) rather than LTP (Schmolesky et al., 2002). In this respect it should be noted that LTP of strong CFs has been reported for P5–P9 only (Ohtsuki and Hirano, 2008), a postnatal age at which CFs do contact the somata of PNs only (Hashimoto et al., 2009). Employing live STED microscopy (Chéreau et al., 2015) might help addressing the question whether dendritic and somatic synapses CFs depress differently.

Multiple pre- and postsynaptic mechanisms may be involved in the observed easing in PPD. Changes in the vesicular release probability (*p*_ves_) represent a likely mechanism. While a developmental increase in *p*_ves_ has been observed for PF-to-PN synapses (Baur et al., 2015), our data would be compatible with a developmental decrease in *p*_ves_ occurring at strong CF-to-PN synapses (Figure 2E, 4C,D). In addition, maturation of PF-PN synapses accompanies a developmental tightening of influx-release coupling, while our data indicate no changes in coupling (Figure 3). Thus, if a decrease in *p*_ves_ underlies easing of PPD, strong CFs would have to undergo a developmental reduction in molecular priming. This scenario could be tested with multiple-probability fluctuation analysis (Silver et al., 1998) performed on young vs. young adult animals. However, electrotonic attenuation of signals from distal CF contacts may have to be taken into consideration (Silver et al., 1998).

Beside changes in the vesicular release probability, easing in PPD may be explained by an acceleration in presynaptic vesicle replenishment (Dittman and Regehr, 1998), an increased clearance of glutamate from the synaptic cleft (Cathala et al., 2005), or by altered kinetics of postsynaptic receptors, for example, via switching between GluR2-containing and -lacking AMPA receptors (Savtchouk and Liu, 2011). While CF-PN synapses express GluR2-containing AMPA receptors throughout postnatal development (Lachamp et al., 2005), the developmentally regulated expression of AMPA receptor isoforms (Monyer et al., 1991) might differ between somatic and dendritic CF synapses. A shift in the relative proportion of flip- versus flop-containing subunits could substantially contribute to the easing of PPD (Koike et al., 2000).

The molecular mechanisms that govern the differential short-term plasticity of CF synapses remain to be clarified. However, our observation that weak and strong CFs show distinct developmental profiles of their short-term plasticity reconciles seemingly conflicting results from previous studies and poses experimentally challenging questions on synapse-specific synaptic maturation.

## Ethics Statement

This study was carried out in accordance with the recommendations of the EU Directive 2010/63/EU. The protocol was approved by the State Directorate of Saxony, Germany.

## Author Contributions

JE conceived and designed the experiments. CP and SB performed the experiments and analyzed the data. CP and JE wrote the manuscript. All authors reviewed the contents of the manuscript and approved the submitted manuscript.

## Conflict of Interest Statement

The authors declare that the research was conducted in the absence of any commercial or financial relationships that could be construed as a potential conflict of interest.
